# Perceptions and Emotions Toward Diets and Supplements Among Reddit Users With Functional/Dissociative Seizures and Their Caregivers: A Mixed‐Methods Analysis of r/PNESsupport

**DOI:** 10.1002/brb3.71497

**Published:** 2026-05-24

**Authors:** Hamid Abbasi, Niloofar Taheri, Ali Jafari

**Affiliations:** ^1^ Student Research Committee, Department of Clinical Nutrition and Dietetics, Faculty of Nutrition Sciences and Food Technology, National Nutrition and Food Technology Research Institute Shahid Beheshti University of Medical Sciences Tehran Iran; ^2^ Department of Psychiatry and Behavioral Sciences Stanford University Palo Alto California USA; ^3^ Student Research Committee, Department of Community Nutrition, Faculty of Nutrition Sciences and Food Technology, National Nutrition and Food Technology Research Institute Shahid Beheshti University of Medical Sciences Tehran Iran; ^4^ Systematic Review and Meta‐analysis Expert Group (SRMEG) Universal Scientific Education and Research Network (USERN) Tehran Iran

**Keywords:** diet, functional seizures, perception, sentiment, supplement

## Abstract

**Background:**

Functional/dissociative seizures (FDS) resemble epileptic seizures but occur without EEG abnormalities. Affected patients report fatigue and gastrointestinal symptoms, seeking dietary approaches. Yet, nutrition's role in FDS remains poorly understood. Thus, this mixed‐methods study analyzed Reddit comments to explore patients’ dietary perceptions and emotions.

**Methods:**

Comments from r/PNESsupport were collected using specific keywords. Only English‐language comments from inception to September 2025 were included. After short and irrelevant comments were removed, the remaining comments were analyzed using sentiment analysis and reflexive thematic analysis. Analyses were performed using RStudio v2025.05.0 and R v4.3.3.

**Results:**

Thirty‐one comments from 23 users with FDS (UwFDS) were analyzed. The total sentiment score was mildly positive (0.080 ± 0.116). Five themes with 12 subthemes emerged: (1) UwFDS emphasized stable blood sugar, noting hypoglycemia and meal timing as seizure triggers; (2) Ketogenic, low‐carbohydrate, and elimination diets were reported as helpful yet raised concerns about hydration and nutritional adequacy; (3) UwFDS described cannabidiol oil, amino acid supplements, and avoiding artificial sweeteners as complementary measures; (4) Food sensitivities and gastrointestinal discomfort were linked to symptom exacerbation or relief; (5) Nutrition was framed as supporting quality of life alongside medical and psychological care.

**Conclusion:**

The positive sentiment reflects hope and agency rather than metabolic efficacy. Integrating nutritional care within psychologically informed frameworks honors patient experience while preserving FDS's non‐organic nature. Future research should evaluate whether structured nutritional guidance, embedded in psychologically informed care, improves outcomes and explore mechanisms like interoceptive processing and the gut–brain axis.

## Graphical abstract

Users with functional/dissociative seizures (UwFDS) identify hypoglycemia and specific diets as key modifiers of their symptoms. These patient‐driven insights, highlighting a gap in clinical care, underscore the need for integrated nutritional strategies and evidence‐based dietary protocols within psychologically informed  FDS management.

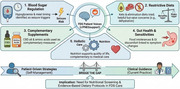

## 1 Introduction

Functional/dissociative seizures (FDS), also previously identified as psychogenic non‐epileptic seizures, represent a neurological disorder marked by seizure‐like events that occur without abnormal findings on electroencephalographic recordings (Hingray et al., 2016; Hingray et al., 2025). These paroxysmal symptoms are frequently associated with disturbances in brain networks (Bodde et al., 2009). Psychological elements, such as unresolved trauma, chronic stress, anxiety, and difficulties in emotional regulation, are recognized as the primary causes of the disorder (Cerasa and Labate, 2018; Labudda et al., 2018). An analytical study estimated the annual incidence of this condition at 3.1 cases per 100,000 individuals, with a prevalence rate of 108.5 cases per 100,000 individuals in the United States (Asadi‐Pooya, 2023). It is important to note that this type of seizure is responsible for 12% to 20% of cases observed in epilepsy clinics (Volbers et al., 2022). Although research has advanced, the underlying mechanisms of this condition are still not obviously understood (Mueller and Szaflarski, 2023).

Current treatment guidelines for FDS emphasize psychological interventions as the cornerstone of management (Goldstein et al., 2020; LaFrance et al., 2014). However, people with FDS often report a range of comorbid physical symptoms, including fatigue, pain, and gastrointestinal issues, which may influence their seizure frequency and overall quality of life (Robson et al., 2018; Thompson et al., 2009). Among these, dietary habits and nutritional intake are frequently discussed by affected patients in community forums as significant factors in their personal symptom management strategies. A growing body of research suggests that diet can impact mental health and neurological functioning through pathways such as gut‐brain axis communication, inflammation, and blood sugar regulation (Bear et al., 2021; Marx et al., 2017). Early clinical investigations support this concept. For instance, a feasibility randomized pilot study executed by Janssen‐Aguilar et al. (Janssen‐Aguilar et al., 2025), in which seventeen outpatients with FDS were randomly assigned to a 6‐week Modified Atkins Diet (MAD) or a control healthy diet, found that while all participants exhibited a decrease in monthly FDS frequency, those on the MAD demonstrated significant improvement in seizures, depression, and anxiety, showing that dietary modification is feasible and could be a helpful complement to FDS treatment. Despite these promising preliminary findings, the role of nutrition in FDS care remains poorly understood and is not routinely addressed in clinical guidelines (Kanemoto et al., 2017; Reilly et al., 2023).

There is a critical gap in grasping the perspective of affected patients on how diet influences FDS. A pilot study executed by Novakova et al. highlighted the need for more research into the lived experiences of individuals with FDS, particularly considering self‐management techniques (Novakova et al., 2019). Understanding these perspectives is essential for developing holistic, patient‐centered care models that address the full spectrum of patient concerns. Online platforms like Reddit have emerged as valuable sources of rich, qualitative data on patient experiences, offering insights into the daily challenges and coping strategies of individuals living with chronic conditions (Biswas and Hasija, 2022).

To address this gap, this mixed‐methods study aimed to explore the perspectives of users with FDS (UwFDS) on the role of nutrition in their routine care. By analyzing discussions from the r/PNESsupport subreddit, this research seeks to illuminate the dietary patterns, strategies, and beliefs held by this patient community. Thus, the findings of this study intend to inform future clinical research and contribute to the development of more comprehensive, integrative care approaches for UwFDS.

## 2 Methods

### 2.1 Study Design

In congruence with the Good Reporting of A Mixed Methods Study (GRAMMS) guideline, this study employed a mixed‐methods design that integrated quantitative and qualitative approaches (O'cathain et al., 2008). Adherence to methodological transparency was ensured in accordance with the Consolidated Criteria for Reporting Qualitative Research (COREQ) guidelines during the qualitative phase's execution and documentation (Tong et al., 2007).

### 2.2 Ethical Considerations

Ethical approval for this study was not secured from the ethics committee of the authors’ division. The ethical framework was guided by established protocols for utilizing internet‐derived data in research (Kaye et al., 2021; Markham and Buchanan, 2012; Adams, 2024). Informed consent was not procured due to the anonymity of Reddit users. However, this approach is generally deemed acceptable since Reddit operates as a public platform where users do not typically have an expectation of privacy (Kaye et al., 2021). Nevertheless, several measures were implemented to safeguard user anonymity and uphold ethical standards. Initially, data were sourced from publicly accessible, moderated subreddits that did not have explicit restrictions against utilizing their content for academic purposes. Additionally, consistent with previous research methodologies, only subreddits with a membership of at least 1000 were included, as participants in these communities are likely to perceive their posts as more public than private. Data were collected without time restrictions, allowing moderators sufficient time to identify and eliminate any personal information. Ultimately, in congruence with established recommendations (McDermott et al., 2013), data cleaning procedures were applied, which involved substituting usernames with participants’ identifiers and omitting dates of comments, subreddit links, and titles.

### 2.3 Data Collection Procedures

**FIGURE 1:**
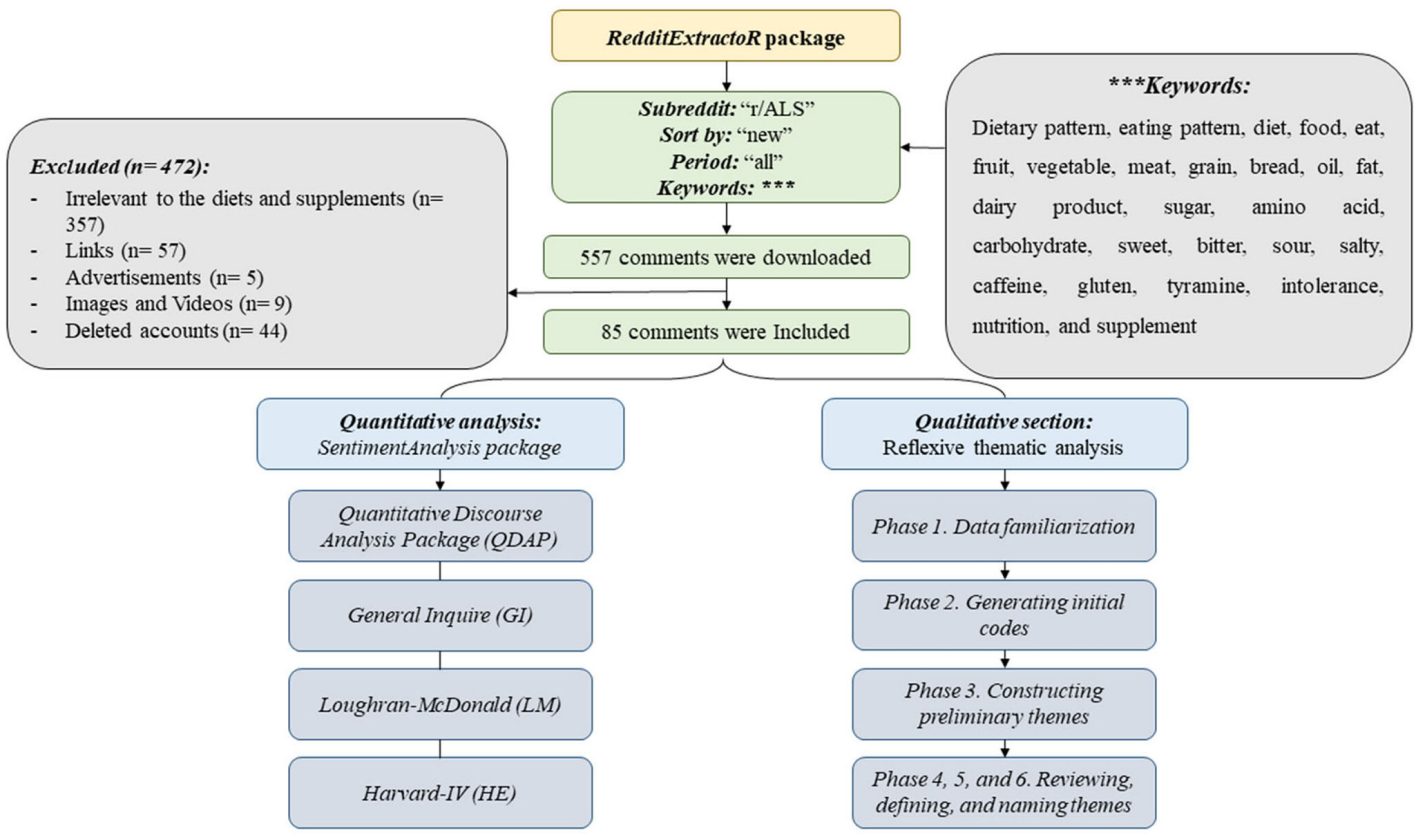
Mixed‐methods study's flow chart.

Comments were collected from the subreddit “r/PNESsupport” utilizing specific search terms (Figure 1). No search filter on Reddit was considered to retrieve threads. A minimal review of these threads was conducted in accordance with several inclusion criteria: (i) the content had to be in English, (ii) it needed to be posted on a moderated public subreddit with a minimum membership of 1000, (iii) the posts should have been made from inception to September 2025, and (iv) they must discuss dietary patterns in relation to “psychogenic nonepileptic seizure,” “functional seizures,” “dissociative seizures,” “nonepileptic attack disorder,” or “functional nonepileptic attacks.” The criteria for exclusion from the analysis were defined as follows: (i) any comments containing hyperlinks, articles, images, or videos were omitted; (ii) comments that consisted of fewer than ten words were excluded; (iii) contributions made by automoderators (automated bots that uphold community regulations on Reddit) or by users whose accounts had been deleted were disregarded (it is noteworthy that if the deleted user was the author of the initial post within a thread, only the comments made after that post were considered); and (iv) comments originating from subreddits that explicitly disallow the use of their content for academic research purposes were also excluded.

### 2.4 Analysis Strategy

The dataset was examined through the lens of reflexive thematic analysis, a qualitative methodology that facilitates the identification of common themes and meanings, such as perceptions and interpretations, within qualitative data (Braun and Clarke, 2019; Varpio et al., 2017). Our analysis employed a hybrid inductive strategy, primarily driven by the data itself while also being informed by existing theoretical frameworks related to dietary habits in individuals experiencing FDS (Braun and Clarke, 2022; Byrne, 2022). Furthermore, we adopted a critical realist perspective in our analysis (Willig, 2013).

### 2.5 Reflexivity Statement

Reflexivity, as a crucial epistemological and methodological practice, necessitates a meticulous examination of how a researcher's unique experiential trajectory and embedded axiological framework fundamentally influence the interpretive paradigms applied to empirical data, thereby shaping the resultant construction and validation of knowledge (Willig, 2013). In undertaking this qualitative study, the first author's (H. A.) positionality as a clinical nutritionist specializing in neurological diseases, coupled with prior work exploring dietary interventions like the ketogenic diet (KD) in FDS (Abbasi, 2025), inherently provides a particular lens through which he approaches this research. While this expertise offers valuable insights into the physiological aspects, the authors acknowledge its potential to subtly influence the interpretation of participant narratives. Therefore, the authors committed to a rigorous reflexive practice, consciously engaging in bracketing to set aside preconceived notions and ensure that the “perceived impacts” genuinely emerged from the participants’ lived experiences, thereby prioritizing their unique perspectives as central to this inquiry.

### 2.6 Analytical Procedure

Thematic analysis, executed reflexively, was guided by Braun and Clarke's six‐phase framework (Braun and Clarke, 2006, 2019). To foster reflexivity, the lead author maintained a dedicated reflective journal. While the primary analysis was spearheaded by the first author (H. A.), both researchers collaboratively engaged in crystallization sessions, a process crucial for enriching understanding, refining data interpretation, and bolstering the credibility of our findings within the broader research landscape (Ellingson, 2009; Tracy, 2010).

### 2.7 Phase 1: Data Familiarization

The initial phase, data familiarization, involved the first author (H. A.) thoroughly reviewing the entire dataset during the cleaning process, while concurrently noting emergent patterns in their reflective journal (Braun and Clarke, 2006).

### 2.8 Phase 2: Generating Initial Codes

Transitioning to the second phase, coding commenced with three authors (H. A., N. T., A. J.) independently coding a randomly selected 10% segment of the dataset, followed by a crystallization meeting where preliminary insights and potential initial codes were discussed. Subsequently, two authors (H. A. and A. J.) proceeded to generate codes for the remaining dataset, primarily focusing on the semantic level, capturing explicit, surface‐level meanings.

### 2.9 Phase 3: Constructing Preliminary Themes

Following a second crystallization meeting dedicated to reviewing these semantic‐level codes, the corresponding authors (H. A. and A. J.) undertook several steps to streamline the code set for subsequent analysis, including collating similar codes, merging redundancies, and discarding those associated with shallow or sparse data. This led into the third phase, theme generation, from which five preliminary themes and subthemes emerged.

### 2.10 Phases 4, 5, and 6: Reviewing, Defining, and Naming Themes

The fourth, fifth, and sixth phases, involving the review, definition, and naming of themes, were facilitated by a third crystallization meeting, where discussions centered on ensuring thematic coherence with the underlying codes and the entire dataset, as well as on selecting names that accurately encapsulated each theme's narrative. Additionally, when comments were mentioned in the Results section, we used comment identification number (CID), upvotes (UV), downvotes (DV), and sentiment score (SS). The dataset of the analyzed comments is provided in the Supplementary Material.

### 2.11 Data Synthesis and Statistical Analysis

Quantitative synthesis and statistical analyses were performed using RStudio v2025.05.0 and R v4.3.3. Specialized software packages were utilized for data collection, comment processing, sentiment analysis, and visualization. The “*RedditExtractoR*” package enabled the identification of discussion threads and the extraction of comments from the r/PNESsupport subreddit through keyword searches (Rivera and Rivera, 2022). Initial data cleaning applied specific exclusion criteria, such as removing non‐comment content, very short comments, and automated posts. Sentiment quantification was conducted with the “*SentimentAnalysis*” package, which implemented four lexicons, encompassing General Inquire (GI), Harvard‐IV (HE), Loughran‐McDonald (LM), and Quantitative Discourse Analysis Package (QDAP) (Feuerriegel et al., 2018). Comment normalization procedures included stopword removal and stemming. All five figures were subsequently created using the “*ggplot2*” package (Wickham, 2016). Normality of total SSs across the five thematic categories was assessed with the Shapiro‐Wilk test as executed by the “*Shapiro.test*” function in R. Accordingly, the scores were reported as mean ± standard deviation.

## 3 Results

### 3.1 Subreddit Data Description

The r/PNESsupport subreddit, comprising 3,800 subscribers, served as the primary data repository for this study. As depicted in Figure 1, the initial retrieval yielded 75 user comments, of which 31 met predefined inclusion criteria for this mixed‐methods study. A total of 44 comments were excluded for various reasons, including 21 comments irrelevant to diets and supplements, 8 containing links, 4 advertisements, 2 containing images and videos, and 9 from deleted accounts. The included comments originated from 23 unique users, indicating diverse participant engagement. The included comments spanned from 2019 to 2025. Analytical metrics demonstrated an average of 2.58 comments per discussion thread and 1.91 distinct users participating per post (Table 1). Additionally, the average total number of words per post were 119.75.

**TABLE 1:** Summary of Subreddit thread retrieval and analytical inclusion.

Characteristics	r/PNESsupport
Subscribers	3800
Comment downloaded	75
Comment included	31
Total numbers of unique commenters	23
Average number of total comments per post	2.58
Average number of total users per post	1.91
Word count per post (excluding title)	119.75

### 3.2 Sentiment Profile of User Comments

**FIGURE 2:**
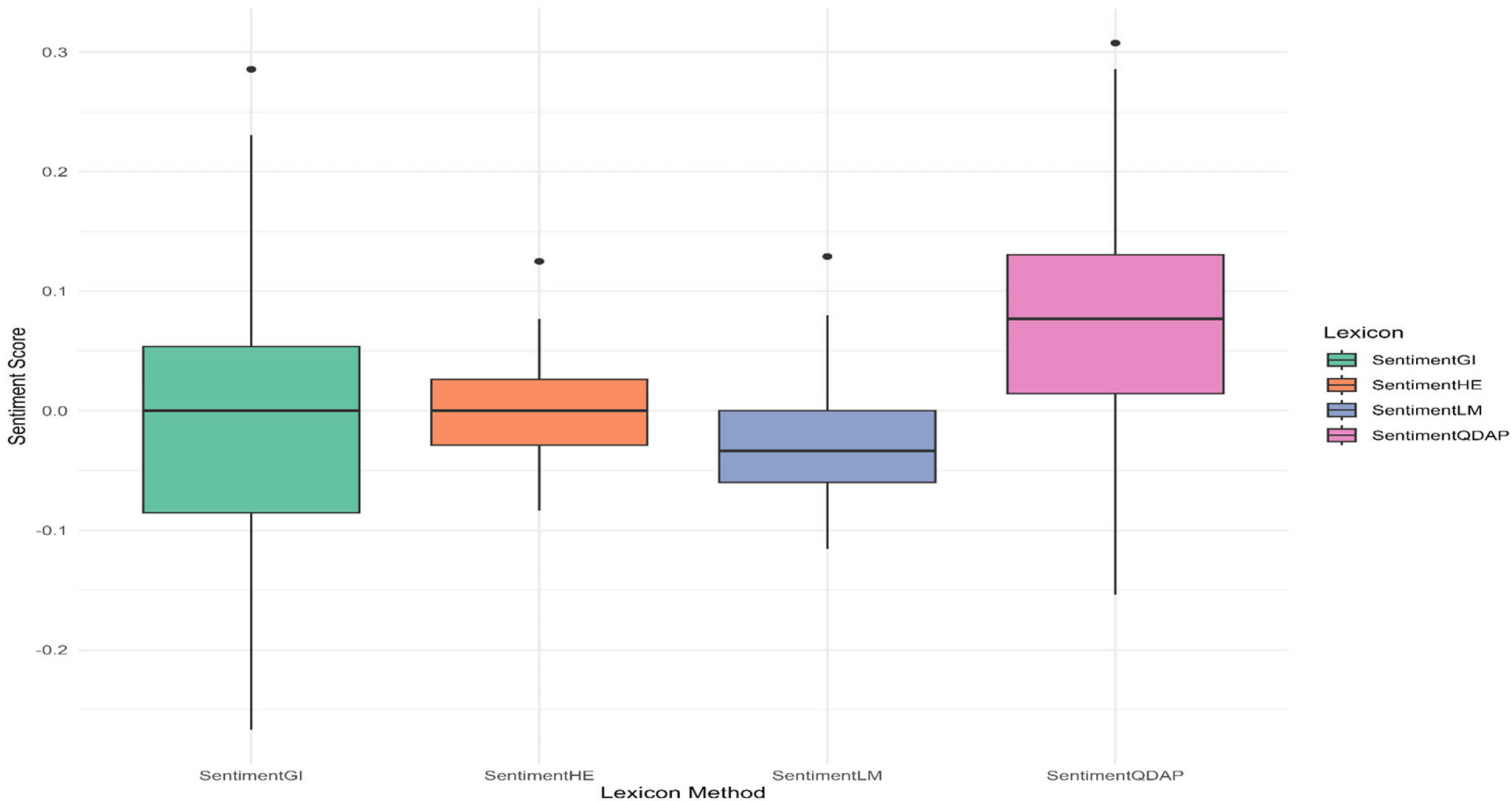
A comparative box plot of sentiment score distributions produced by four various lexicon methods (GI, HE, LM, and QDAP).

**FIGURE 3:**
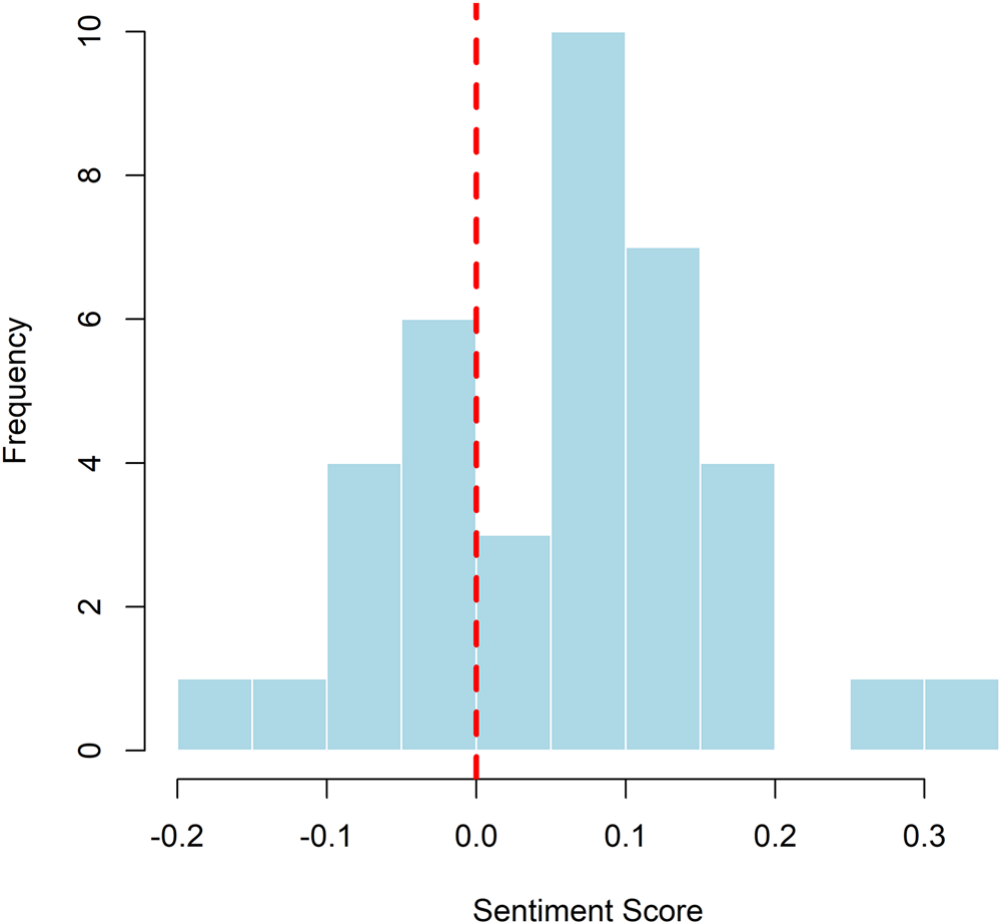
Frequency distribution of net sentiment score calculated using the QDAP lexicon. The vertical dashed line at zero separates negative scores from positive scores.

**FIGURE 4:**
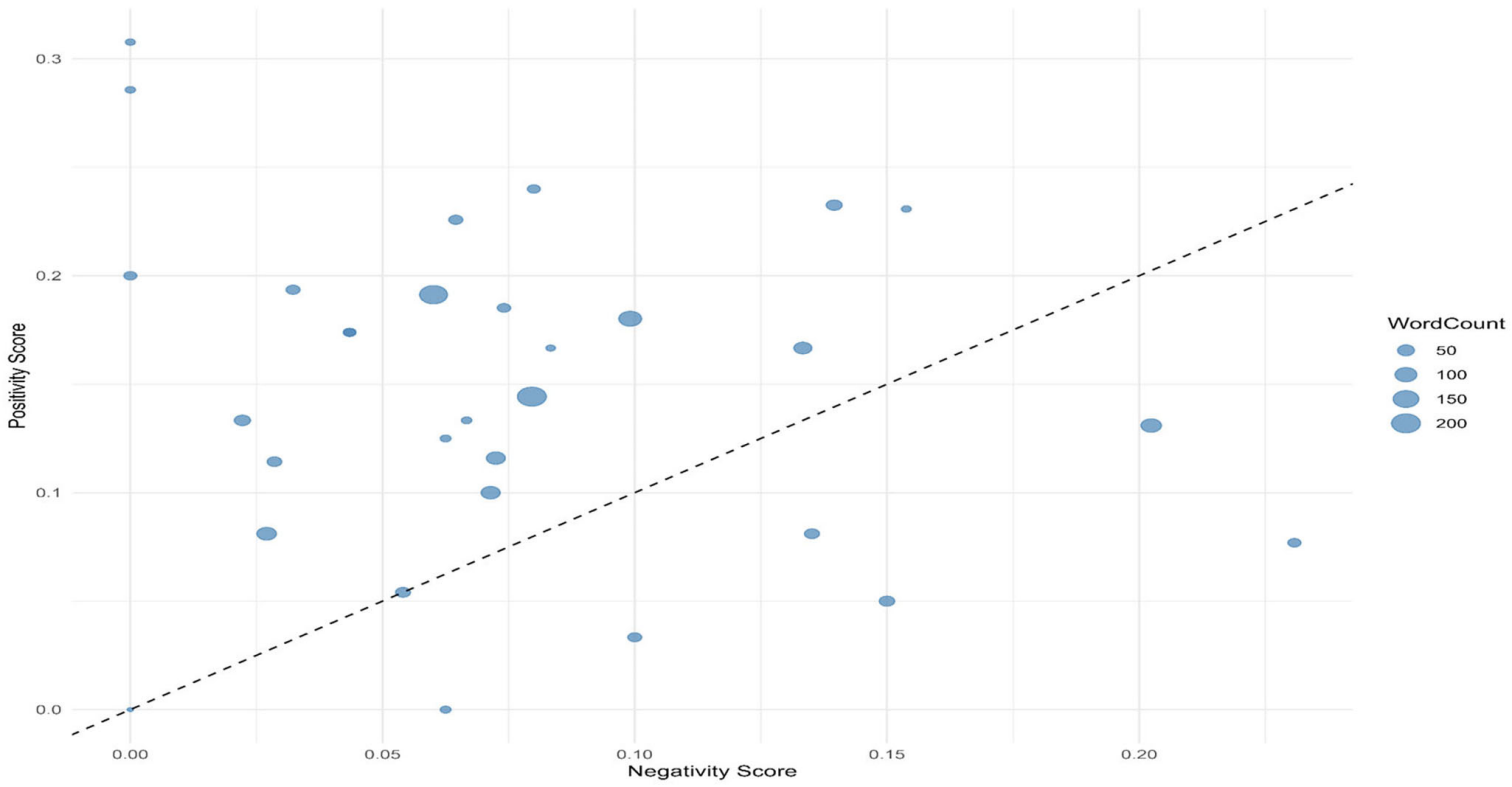
A scatter plot comparing the positivity and negativity scores of each comment. The dashed line indicates where positivity and negativity scores are equal.

**FIGURE 5:**
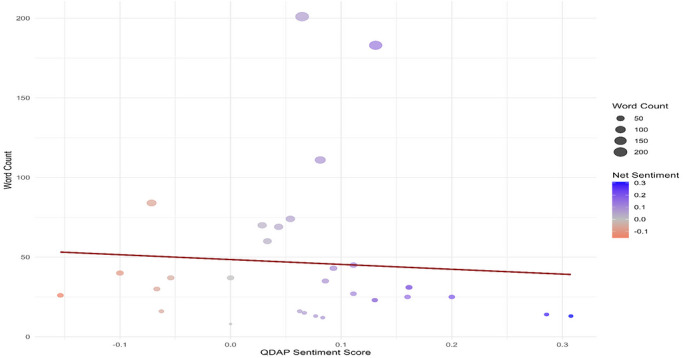
A scatter plot illustrating the relationship between word count and QDAP sentiment score.

**FIGURE 6:**
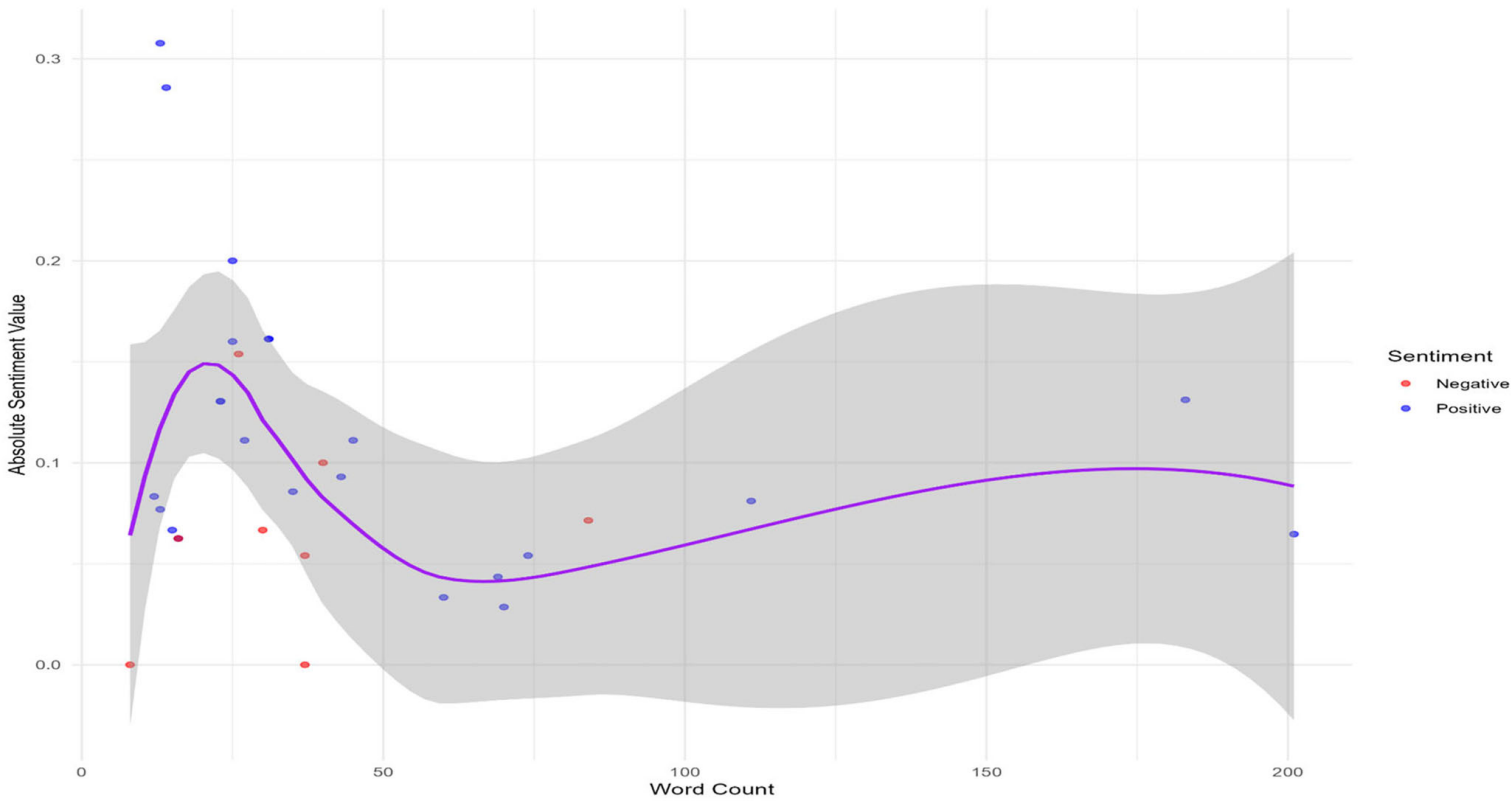
The relationship between word count and absolute sentiment intensity.

The sentiment analysis of the collected comments indicates a generally positive leaning. Evaluation across four separate sentiment groups utilizing four distinct lexicons showed that the QDAP lexicon consistently yielded the highest SSs, suggesting it is the most attuned to positive expressions (Figure 2). The distribution of net SSs is positively skewed, with the most frequent values occurring between 0.1 and 0.3 (Figure 3). Further scrutiny confirms that for a majority of comments, the positivity score exceeds the negativity score underscoring the overall favorable sentiment (Figure 4). Interestingly, a weak negative correlation was detected between the length of a comment and its QDAP SS, indicating that longer comments are slightly more likely to have a less positive sentiment (Figure 5). Analysis of sentiment intensity demonstrates a clear pattern with the strongest sentiments present in the shortest comments. The intensity diminishes as comment length increases, eventually stabilizing for longer texts (Figure 6).

### 3.3 Qualitative Evaluation

In this mixed‐methods study, we categorized the included comments into five primary themes with 12 subthemes, as mentioned below. Moreover, Table 2 presents the thematic constructs, classification of subthemes, and supporting comments. The total SS for each theme is presented in Table 3.

**TABLE 2:** Summary of emergent thematic constructs, subtheme classifications, and evidentiary quotations.

Theme	Subtheme	Exact comment
**Eating patterns and blood sugar regulation**	**Hypoglycemia as a reported seizure trigger**	“Since you mentioned you haven't been eating much, I'd honestly lean more towards it being hypoglycemia. The symptoms of hypoglycemia really do fit what you have listed to a T. I'd suggest getting a glucometer and check your glucose levels when you feel off and haven't eaten. I have both PNES and diabetes and I have had hypoglycemia issues since I was a child. Also keep in mind that you can have hypoglycemia episodes without being diabetic.”
		“Hi! I'm diabetic, I had felt what op described only once…I literally crawl to a store and beg for something sweet. The whole deal probably lasted like 2 h…I have hyper though, not hypo. Can it be possible in my case?”
		“After the orange juice I felt better (I was able to feel my legs normal and I was able to see clearly in that moment) but I don't remember anything else in those two hours except those few details…”
		“Last you might just be high anxiety and stressing yourself out unintentionally…But rest assured it's common and you are not dying or anything, just a bump in the road you need to look into. Best of luck, take care and please try to EAT! Apple sauce, yogurt, granola bars…easy snacks. Get glucose sugar pills too if needed and drink Ensure vitamin drinks. No food or water and your brain and body will break down fast.”
	**Timing of meals and hypoglycemia**	“Not so much a change in diet, but it helped me to eat sooner in the day to avoid low blood sugar levels, which in turn had helped my symptoms.”
	**Disordered eating patterns and reported seizure frequency**	“I know those sounds stupid to many but I also had anorexia and I still though recovered don't eat all that great and I get hypos now if I don't eat a certain amount of sugar by a certain time of waking up and before bed. So maybe try having a carb before bed and something sugary in the morning. Hypos cause seizures in me.”
		“Yes, digestive issues trigger me to collapse and have loss of muscle/motor control. Low blood sugar can trigger seizures. I hate food and eating in general, so it's safe to say I have a disordered relationship with food, and that plays a role in my symptoms.”
**Restrictive and structured diets**	**Ketogenic and low‐carbohydrate approaches**	“One of the first things that cut the seizures down in amount was going keto. I did that for three years but I was hard on my body and I'm no longer keto. I still limit my sugar intake if I'm have a rough spell because I know highs and lows are best avoided.”
		“Hi, I tried low carb and it's working really well for me. I think the constant blood sugar is less stress for the system. Also drinking enough (water) is really important to avoid attacks. Low carb also helps to brighten the mood and makes me psychologically feel better.”
		“I've heard of keto being helpful but my concerns are how dehydration and lack of good diet effects the body. I've talked to my therapist about this. I think it's important to drink water because exhaustion can be a trigger for some ppl and missing a meal is also dangerous because pain ‘feeling low energy’ is exhausting to the body.”
		“I've been wondering whether the keto diet would help PNES as well…I quit drinking coffee or consuming any caffeine and eating gluten and I feel so much better now. I have fewer migraines and since I stopped caffeine I haven't had seizure in over a year now.”
		“I've been on keto‐light (under 100 g carbs/day) for about a year for unrelated health problems and I have noticed reduced seizures in that time, but I'm not sure if it's because of my dietary changes or not.”
		“It did not work for me. I had a Dr. recommend. I will advise not going through with it unless your Dr. suggests or talking to a dietitian first.”
	**Elimination diets (caffeine, gluten, tyramine, sugar, and oil)**	“My neurologist is the one who originally brought it up because I had an ER visit in relation to accidentally eating too much tyramine…The test for me to figure out if it was related was basically a low tyramine elimination diet…”
		“Spicy food can activate mast cells due to the capsaicin and cause allergic symptoms, so maybe that would be why for you. For me it's (probably) tyramine…”
		“I quit drinking coffee or consuming any caffeine and eating gluten and I feel so much better now…Since I stopped caffeine I haven't had a seizure in over a year now.”
		“I always felt that mine were triggered by high amounts of sugar and oil.”
	**Portion sizes and overeating**	“My portion sizes have dropped since eating a lot can be triggering for me. We even got portion plates from Walmart so I don't go overboard.”
**Complementary and alternative nutrition strategies**	**Cannabidiol oil**	“CBD oil. I started taking it around 2 months ago and I went from 3–5 seizures a week to 2 in the last month. That stuff works magic. Of course consult your PCP before beginning it, but in the end I believe the benefits are too great to not at least try it.”
		“We've read in this but she's concerned over a drug test at work. I've read conflicting information on this. Any personal insight?”
		“You can get non‐THC CBD. It doesn't show on a drug test.”
	**Amino acid and neurotransmitter supplements**	“Thank you so much for sharing your experiences and research…Hmm don't have any L‐tryptophan on hand but I do have some 5‐HTP so hopefully that will have the same effect until I can get to a pharmacy.”
		“Ah, 5‐HTP made me throw up and it apparently doesn't convert into kynurenine, but instead mostly serotonin. L‐tryptophan is prescription only in Canada.”
		“I'd recommend cutting out artificial sugars. There is a link between how out body processes them and our nervous system. It's helped to reduce the intensity of mine. Also, highly recommend GABA supplements. I take 200 mg a day, and it helps to keep them manageable to function every day.”
**Food sensitivities and gut health**	**Food intolerance and immune reactions**	“Good question, I would advise to check food intolerances as well as cut all processed sugar. There are neuropsychiatrist and also food psychiatrics, I recently learned about that…”
		“I mean, I think if you have food sensitivities and ignore them (i.e., Lactose intolerance doesn't stop you eating cheese) or if you have an unhealthy diet it definitely won't help, but that's true of everything.”
	**Gastrointestinal triggers**	“Worked on getting my gastrointestinal issues in a better place. That has also helped.”
		“I definitely do find there is a link to my gut health. It's linked to our natural biology. Hope your find a balance of food and supplements that works for you.”
**Nutrition for symptom relief and coordinated care**	**Optimizing quality of life and symptom management**	“I think diet influences every part of our lives. Yet, I don't believe it is the cause or cure all to the reactions happening in our nervous system. A healthy balanced diet based on nutrition will help with quality of life and symptom management. Symptoms don't disappear with a trend diet.”
	**Integration with psychological and medical care**	“I went to my neuro and nutritionist.”
		“All good points, I agree on exhaustion being a trigger. I think my biggest issue currently is needing to improve my self‐care habits.”

**TABLE 3:** Descriptive statistics of total sentiment scores for the five thematic categories derived from UwFDS comments.

Theme number	Comments	Minimum	Maximum	Mean	Standard Deviation
1	7	−0.153	0.161	0.029	0.101
2	11	−0.100	0.403	0.113	0.141
3	6	0.000	0.200	0.068	0.087
4	4	−0.054	0.285	0.101	0.140
5	3	0.000	0.125	0.072	0.064
Total	31	−0.153	0.403	0.080	0.116

### 3.4 Theme 1: Eating Patterns and Blood Sugar Regulation

#### 3.4.1 Subtheme 1.1: Hypoglycemia as a Reported Seizure Trigger

Several participants described hypoglycemia as a trigger for their seizures. They also reported episodes of weakness, confusion, and fear that could be compatible with hypoglycemia. Participants mentioned nutritional imbalances as both a background health concern and an immediate contributor to seizure vulnerability.

*“Since you mentioned you haven't been eating much, I'd honestly lean more towards it being hypoglycemia. The symptoms of hypoglycemia really do fit what you have listed to a T. I'd suggest getting a glucometer and check your glucose levels when you feel off and haven't eaten. I have both FDS and diabetes and I have had hypoglycemia issues since I was a child. Also keep in mind that you can have hypoglycemia episodes without being diabetic. (CID:2, UV:3, DV:0, SS:0)”*

One participant had difficulty distinguishing between panic and hypoglycemia. Their memory gaps and bodily distress showed how strongly nutritional states can overlap with psychological symptoms, complicating self‐understanding and care‐seeking.

*“Hi! I'm diabetic; I had felt what op described only once… I literally crawl to a store and beg for something sweet. The whole deal probably lasted like 2 h… I have hyper though, not hypo. Can it be possible in my case? (CID:2_1, UV:2, DV:0, SS:‐0.032)”*

Another respondent noted how recovery after consuming juice clarified the episode as likely blood sugar‐related. Such reflections highlight the trial‐and‐error strategies participants use to parse the role of diet in FDS.

*“After the orange juice I felt better (I was able to feel my legs normal and I was able to see clearly in that moment) but I don't remember anything else in those two hours except those few details… (CID:2_1_1_1UV:2, DV:0, SS:0.161)”*

Some individuals linked skipped meals or insufficient snacks to worsening symptoms. Their advice to others emphasized small, accessible foods as protective strategy.

*“Last you might just be high anxiety and stressing yourself out unintentionally…But rest assured it's common and you are not dying or anything, just a bump in the road you need to look into. Best of luck, take care and please try to EAT! Apple sauce, yogurt, granola bars…easy snacks. Get glucose sugar pills too if needed and drink Ensure vitamin drinks. No food or water and your brain and body will break down fast. (CID:4, UV:1, DV:0, SS:0.081)”*

#### 3.4.2 Subtheme 1.2: Timing of Meals and Hypoglycemia

A few participants explained how eating earlier in the day helped regulate their symptoms. Their accounts suggest that not just what is eaten but when food is consumed can make a tangible difference.

*“Not so much a change in diet, but it helped me to eat sooner in the day to avoid low blood sugar levels, which in turn had helped my symptoms. (CID:2, UV:1, DV:0, SS:0.066)”*

#### 3.4.3 Subtheme 1.3: Disordered Eating Patterns and Reported Seizure Frequency

The intersection of FDS with past eating disorders or disordered patterns of intake appeared significant. For some, restrictive eating intensified the likelihood of possible hypoglycemic episodes, which they recognized as seizure triggers.

*“I know those sounds stupid to many but I also had anorexia and I still though recovered don't eat all that great and I get hypos now if I don't eat a certain amount of sugar by a certain time of waking up and before bed. So maybe try having a carb before bed and something sugary in the morning. Hypos cause seizures in me. (CID:2, UV:2, DV:0, SS:0.085)”*

Others described a strained relationship with food in general, stating that their dislike of eating or poor eating patterns heightened their seizure risk. This illustrates how nutritional vulnerability in FDS may be exacerbated by ambivalence toward food.

*“Yes, digestive issues trigger me to collapse and have loss of muscle/motor control. Low blood sugar can trigger seizures. I hate food and eating in general, so it's safe to say I have a disordered relationship with food, and that plays a role in my symptoms. (CID:3, UV:2, DV:0, SS:−0.153)”*

### 3.5 Theme 2: Restrictive and Structured Diets

#### 3.5.1 Subtheme 2.1: Ketogenic and Low‐Carbohydrate Approaches

Some participants found that KDs provided meaningful benefits, such as reducing seizure frequency or controlling sugar fluctuations. However, these experiences were often paired with challenges, such as difficulty maintaining the diet or physical strain over time.

*“One of the first things that cut the seizures down in amount was going keto. I did that for 3 years but I was hard on my body and I'm no longer keto. I still limit my sugar intake if I'm have a rough spell because I know highs and lows are best avoided. (CID:2, UV:6, DV:0, SS:0.064)”*

Others explained positive outcomes from low‐carbohydrate approaches, particularly in relation to energy stability and mood. The steady regulation of blood sugar and adequate hydration were considered essential components that made the diet supportive of both physical and psychological well‐being.

*“Hi, I tried low carb and it's working really well for me. I think the constant blood sugar is less stress for the system. Also drinking enough (water) is really important to avoid attacks. Low carb also helps to brighten the mood and makes me psychologically feel better. (CID:4, UV:2, DV:0, SS:0.161)”*

Concerns were also raised about potential risks of ketogenic or restrictive diets. Participants highlighted the possibility of dehydration, missed meals, and poor nutritional balance. Some emphasized that these issues can intensify exhaustion which may itself serve as a trigger.

*“I've heard of keto being helpful but my concerns are how dehydration and lack of good diet effects the body. I've talked to my therapist about this. I think it's important to drink water because exhaustion can be a trigger for some ppl and missing a meal is also dangerous because pain “feeling low energy” is exhausting to the body. (CID:2, UV:2, DV:0, SS:0.033)”*

UwFDS considered whether ketogenic strategies could extend to other conditions or lifestyle changes. In some cases removing additional dietary triggers such as caffeine or gluten was reported as beneficial, leading to noticeable reductions in seizures and migraines.

*“I've been wondering whether the keto diet would help FDS as well…I quit drinking coffee or consuming any caffeine and eating gluten and I feel so much better now. I have fewer migraines and since I stopped caffeine I haven't had seizure in over a year now. (CID:6, UV:1, DV:0, SS:0.054)”*

Another participant practiced moderate forms of the diet such as limiting carbohydrates without adopting a full ketogenic plan. In these cases, there was uncertainty about whether the dietary change directly caused improvements or whether other factors may have contributed.

*“I've been on keto‐light (under 100 g carbs/day) for about a year for unrelated health problems and I have noticed reduced seizures in that time, but I'm not sure if it's because of my dietary changes or not. (CID:2, UV:3, DV:0, SS:0.16)”*

Ultimately, one participant reported a lack of success with ketogenic dieting and emphasized the importance of professional guidance. They highlighted that individuals should consult a doctor or dietitian before committing to such approaches.

*“It did not work for me. I had a Dr. recommend. I will advise not going through with it unless your Dr. suggests or talking to a dietitian first. (CID:1, UV:3, DV:0, SS:0.307)”*

#### 3.5.2 Subtheme 2.2: Elimination Diets (caffeine, gluten, tyramine, sugar, and oil)

UwFDS explained how dietary changes provided them with relief. They explained that eliminating certain foods, like tyramine reduced both migraine episodes and seizure activity. While results varied, the process of testing these diets gave them greater insight into their own health.

*“My neurologist is the one who originally brought it up because I had an ER visit in relation to accidentally eating too much tyramine… The test for me to figure out if it was related was basically a low tyramine elimination diet… (CID:1_1, UV:4, DV:0, SS: 0.043)”*

Another user with FDS shared observations about how food components can influence bodily reactions. They noted that particular substances, such as capsaicin in spicy foods, could trigger allergic symptoms through mast cell activation. For another participant, tyramine appeared to be the main trigger.

*“Spicy food can activate mast cells due to the capsaicin and cause allergic symptoms, so maybe that would be why for you. For me it's (probably) tyramine… (CID:2_1, UV:5, DV:0, SS:‐0.1)”*

Caffeine and gluten were also considered strong contributors to neurological symptoms. Eliminating them was described as producing a noticeable improvement in overall well‐being and, in one case, stopping seizures entirely for over a year.

*“I quit drinking coffee or consuming any caffeine and eating gluten and I feel so much better now… Since I stopped caffeine I haven't had a seizure in over a year now. (CID6, UV1, DV0, SS:0.054)”*

Another uwith FDS further identified sugar and oil as personal triggers. They felt that high intake of these substances directly worsened their symptoms, reinforcing the importance of dietary monitoring and adjustment.

*“I always felt that mine were triggered by high amounts of sugar and oil. (CID:4, UV:1, DV:0, SS:0)”*

#### 3.5.3 Subtheme 2.3: Portion Sizes and Overeating

A few noted that eating large amounts at once could trigger episodes. Using portion‐control strategies, such as specific plates, helped them manage intake and avoid destabilizing symptoms.

*“My portion sizes have dropped since eating a lot can be triggering for me. We even got portion plates from Walmart so I don't go overboard. (CID:1, UV:2, DV:0, S:0.111)”*

### 3.6 Theme 3: Complementary and Alternative Nutrition Strategies

#### 3.6.1 Subtheme 3.1: Cannabidiol Oil

Cannabidiol (CBD) oil was highlighted as a nutritional supplement that brought a reduction in seizure frequency. Its use, however, raised concerns about workplace drug testing, showing how practical barriers intersect with nutritional experimentation.

*“CBD oil. I started taking it around 2 months ago and I went from 3–5 seizures a week to 2 in the last month. That stuff works magic. Of course consult your PCP before beginning it, but in the end I believe the benefits are too great to not at least try it. (CID:2, UV:2, DV:0, SS:0.2)”*

*“We've read in this but she's concerned over a drug test at work. I've read conflicting information on this. Any personal insight? (CID:2_1, UV:1, DV:0, SS:0.083)”*

*“You can get non‐THC CBD. It doesn't show on a drug test. (CID:2_1_1, UV:2, DV:0, SS:0)”*

#### 3.6.2 Subtheme 3.2: Amino Acid and Neurotransmitter Supplements

Several participants experimented with dietary supplements tied to neurotransmitter regulation. Tryptophan, serotonin, and gamma‐aminobutyric acid (GABA) were discussed as possible means to reduce seizure activity, although results varied across experiences.

*“Thank you so much for sharing your experiences and research… Hmm, don't have any L‐tryptophan on hand but I do have some 5‐HTP so hopefully that will have the same effect until I can get to a pharmacy. (CID:2, UV:2, DV:0, SS:0.028)”*

One participant shared that 5‐hydroxytryptophan (5‐HTP) caused them discomfort and explained that it does not convert into kynurenine but instead primarily into serotonin. They also pointed out that in Canada, L‐tryptophan requires a prescription, making access more limited.

*“Ah, 5‐HTP made me throw up and it apparently doesn't convert into kynurenine, but instead mostly serotonin. L‐tryptophan is prescription only in Canada. (CID:2_1, UV:2, DV:0, SS:0)”*

Another participant emphasized the importance of lifestyle changes alongside supplements. They suggested avoiding artificial sugars due to the way they affect the nervous system. They also recommended daily use of GABA, noting that a dosage of 200 mg helped reduce seizure intensity and supported day‐to‐day functioning.

*“I'd recommend cutting out artificial sugars. There is a link between how our body processes them and our nervous system. It's helped to reduce the intensity of mine. Also, highly recommend GABA supplements. I take 200 mg a day, and it helps to keep them manageable to function every day. (CID:3, UV:3, DV:0, SS:0.115)”*

### 3.7 Theme 4: Food Sensitivities and Gut Health

#### 3.7.1 Subtheme 4.1: Food Intolerance and Immune Reactions

Food intolerances were seen as potential contributors to the onset of FDS. Spicy foods, processed sugars, and other sensitivities were described as activating symptoms through immune or metabolic pathways.

*“Good question, I would advise to check food intolerances as well as cut all processed sugar. There are neuropsychiatrists and also food psychiatrics; I recently learned about that… (CID:1, UV:3, DV:0, SS:0.111)”*

Another affected individual emphasized that ignoring food sensitivities could worsen health outcomes. For instance, continuing to eat cheese despite lactose intolerance or maintaining an unhealthy diet may contribute to the persistence of symptoms.

*“I mean, I think if you have food sensitivities and ignore them (i.e., Lactose intolerance doesn't stop you eating cheese) or if you have an unhealthy diet it definitely won't help, but that's true of everything. (CID:5, UV:1, DV:0, SS:‐0.054)”*

#### 3.7.2 Subtheme 4.2: Gastrointestinal Triggers

Gut discomfort and wider gastrointestinal challenges were often described as making FDS more arduous to manage. Participants suggested that when they focused on improving their digestion, they noticed a reduction in seizure frequency.

*“Worked on getting my gastrointestinal issues in a better place. That has also helped. (CID:2, UV:6, DV:0, SS: 0.064)”*

Another participant also recognized a strong link between gut health and overall well‐being in UwFDS. They felt that finding the right balance in diet and supplements was an important step in managing their condition more effectively.

*“I definitely do find there is a link to my gut health. It's linked to our natural biology. Hope your find a balance of food and supplements that works for you. (CID:1, UV:2, DV:0, SS:0.285)”*

### 3.8 Theme 5: Nutrition for Symptom Relief and Coordinated Care

#### 3.8.1 Subtheme 5.1: Optimizing Quality of Life and Symptom Management

Many participants expressed that diet could support symptom management and improve quality of life but should not be seen as a cure. They stressed the importance of balance, hydration, and sustainable eating practices rather than extreme diets.

*“I think diet influences every part of our lives. Yet, I don't believe it is the cause or cure all to the reactions happening in our nervous system. A healthy balanced diet based on nutrition will help with quality of life and symptom management. Symptoms don't disappear with a trend diet. (CID:1, UV:2, DV:0, SS:0.093)”*

#### 3.8.2 Subtheme 5.2: Integration With Psychological and Medical Care

The broader narrative showed that participants did not view nutrition in isolation. They consistently tied dietary changes to therapy, medication, and psychological well‐being. For them, nutrition was a complementary strategy rather than a stand‐alone solution.

*“I went to my neuro and nutritionist. (CID:2, UV:1, DV:0, SS:0)”*

*“All good points; I agree on exhaustion being a trigger. I think my biggest issue currently is needing to improve my self‐care habits. (CID:2_1, UV:2, DV:0, SS:0.125)”*

## 4 Discussion

This mixed‐methods analysis of 31 comments from 23 unique UwFDS on the r/PNESsupport subreddit identified five themes concerning dietary triggers, restrictive diets, supplement use, food sensitivities, and nutrition as complementary care. Quantitative sentiment analysis using the QADP lexicon revealed an overall score of 0.080 ± 0.116. This consistently positive affective tone, however, must not be misconstrued as evidence of direct metabolic effects of nutrition on FDS. Rather, it reflects the psychological uplift that accompanies active self‐management, social validation, and the restoration of perceived control in the face of a poorly understood neurological condition.

### 4.1 Theme 1: Hypoglycemia as a Perceived Seizure Trigger

UwFDS who attributed seizure episodes to low blood sugar or missed meals expressed a weakly positive sentiment of 0.029 ± 0.101. We found it notable that this value hovered near neutrality; one might anticipate stronger positive affect if these individuals were describing a reliably modifiable biological trigger. FDS, by their very nature, arise in the absence of epileptiform electroencephalographic disturbance (Hingray et al., 2025) and are not propelled by the neuronal hyperexcitability that defines hypoglycemic convulsions in epilepsy (Perez et al., 2015). It strikes as implausible, therefore, that the sentiment captured here reflects a direct metabolic pathway. A more coherent account positions the somatic discomfort for hypoglycemia, manifested as tremor, mental fog, and autonomic surge, as an interoceptive stressor that, in psychologically vulnerable UwFDS, ignites FDS via anxiety amplification and conditioned hypervigilance (Asadi‐Pooya et al., 2024; Kanner et al., 2012). The faint positivity we observed likely springs from the solace of having a tangible action plan (e.g., carrying snacks and checking glucose) rather than from any true modification of seizure physiology.

### 4.2 Theme 2: Restrictive and Structured Diets

Comments detailing experimentation with ketogenic, low‐carbohydrate, and elimination diets generated the highest sentiment across all themes (0.113 ± 0.141). This pronounced positivity seems incongruent with the modest evidence of a cure; we would not necessarily expect the accompanying discourse to be so buoyant, given the well‐documented hardships of dietary restriction (Janssen‐Aguilar et al., 2025). We interpret this enthusiasm as a psychological dividend of reclaiming agency. The act of adhering to a structured nutritional protocol offers UwFDS a concrete framework for managing a condition that often feels chaotic and beyond personal influence (Laurent et al., 2024). Moreover, the behavioral activation inherent in self‐monitoring food intake may secondarily lift mood and dampen anxiety (Tate et al., 2020). UwFDS themselves acknowledged tangible drawbacks, dehydration, and nutritional gaps, yet the net sentiment remained robustly positive, a testament to the psychological value of active engagement. We emphasize that this emotional benefit must not be conflated with evidence of a direct anticonvulsant mechanism. The structured nature of dietary interventions may reduce overall stress, provide a sense of control, or serve as a form of behavioral activation that addresses underlying mood disturbances rather than altering seizure pathophysiology directly. The KD and carbohydrate‐restricted diets, when sustained, have been shown in other populations to improve psychological well‐being, quality of life, and mood (Laurent et al., 2024; Sindler et al., 2024) and, epidemiologically, are linked to lower risks of depression and anxiety (Qin et al., 2025). Furthermore, individuals following KD for mental health often report enhanced self‐esteem, motivation, and a renewed sense of purpose (Qin et al., 2025), underpinning the view that the positive sentiment we observed is predominantly psychologically driven. Hence, the enthusiasm of UwFDS for restrictive diets, though psychologically understandable, must be meticulously distinguished from a direct anticonvulsant effect on FDS.

### 4.3 Theme 3: Complementary and Alternative Nutrition Strategies

Discussions centered on CBD oil, GABA, and amino acid supplements, and associated nutraceuticals carried a moderately positive sentiment of 0.068 ± 0.087. Although several UwFDS recounted subjective reductions in seizure burden, it seems there is no rigorous evidence establishing that these substances directly modulate the neuronal circuits responsible for FDS. The positivity strikes us as a manifestation of therapeutic hope and the placebo‐adjacent reassurance that accompanies taking proactive steps toward wellness (Novakova et al., 2019). For UwFDS who have endured years of diagnostic delay and restricted treatment options, the simple act of self‐administering a supplement may confer a meaningful sense of control and diminish illness‐associated distress. The diagnostic journey for FDS averages 7 to 10 years (Dworetzky, 2014), during which many individuals receive ineffective treatments for presumed epilepsy, fostering a persistent search for alternative explanations (Pretorius, 2016). The complete absence of negative sentiment in this theme reinforces our view that the emotional tone is propelled by psychological forces, such as optimism, peer endorsement, and the comfort of action, rather than by objective pharmacodynamic effects.

### 4.4 Theme 4: Food Sensitivities and Gut Health

UwFDS, who linked symptom exacerbation to specific foods, encompassing tyramine, capsaicin, caffeine, and gluten, expressed a sentiment of 0.101 ± 0.140, even though individuals’ comments ranged into negative territory (minimum: −0.054). This coexistence of positive and negative affect reflects, in our view, the ambivalent reality of living with unpredictable somatic experiences. The notion that these dietary compounds exert a direct pathophysiological influence in FDS lacks credible support. We found it far more plausible that gastrointestinal discomfort or the perception of food intolerance operates as a potent interoceptive stressor, engaging autonomic and emotional pathways that lower the threshold for functional symptom emergence (Hyman, 2021; Jansson‐Knodell et al., 2022). The positive sentiment may arise from the relief of identifying a suspected trigger, whereas negative sentiment captures the genuine distress of bodily unpredictability. Crucially, neither valence offers substantiation of a metabolic or immunologic mechanism in FDS.

### 4.5 Theme 5: Nutrition as Complementary Care

UwFDS consistently portrayed nutritional strategies as adjunctive supports for quality of life rather than definitive cures, yielding a SS of 0.072 ± 0.064. This moderate positivity aligns comfortably with the psychologically mediated framework we have advanced. UwFDS did not claim that dietary changes abolished their seizures; they emphasized instead that stable nourishment, adequate hydration, and mindful eating mitigated overall stress and enhanced daily function. Such expectations resonate with evidence‐based treatment tenets that prioritize psychological therapies for FDS (L. H. Goldstein et al. 2020). The sentiment expressed here does not clash with mechanistic understanding. It reflects the genuine, albeit indirect, benefits of nutritional stability on systemic well‐being, which may ease the burden of functional symptoms by attenuating psychological distress. Clinicians managing FDS must navigate a fine line: on one hand, dismissing dietary concerns may risk damaging the therapeutic alliance; on the other hand, endorsing organic explanations may increase the risk of reinforcing illness beliefs that are inconsistent with the functional nature of the disorder.

### 4.6 Integrating Sentiment Profile and Clinical Implications

The consistently positive mean SSs across all themes, irrespective of mechanistic plausibility, indicated that UwFDS extracted considerable psychological comfort from engaging with nutritional concepts. This observation carries actionable clinical weight. We urge providers to validate UwFDS nutritional narratives without endorsing an organic etiology, to deliver psychoeducation on the psychologically elevated prevalence in this population, and to facilitate referrals to registered dietitians conversant with functional neurological disorders.

### 4.7 Strengths, Limitations, and Future Directions

The current patient perspective data in this underexplored area fills a critical evidence gap, particularly given the limited research on nutritional factors in FDS compared to epileptic seizures. The critical realist epistemological framework and structured reflexivity practices, including the lead author's maintained reflective journal, enhanced credibility and transparency while acknowledging researcher positionality as a clinical nutritionist specializing in neurological conditions (Willig 2013). Real‐world naturalistic data reflected authentic patient experiences often absent from controlled clinical settings, revealing patient agency and self‐management strategies that inform clinical practice development.

The exclusive reliance on English‐language data from a single subreddit introduces inherent selection bias, limiting generalizability to non‐English speakers, individuals without internet access, and those utilizing different support networks. The relatively small number of comments (31 comments from 23 users), while appropriate for qualitative exploration, precludes robust quantitative generalizations and may not capture the full spectrum of nutritional experiences among individuals with FDS. Self‐report limitations and potential recall bias could not be verified through direct clinical assessment or biomarker validation. Missing perspectives from certain demographic subgroups, particularly older adults and those from diverse socioeconomic backgrounds, may limit the applicability of findings to broader FDS populations. Another limitation of this study was the use of the Reddit platform alone, which limits generalizability to the broader FDS population. The inability to distinguish between patient and caregiver posts in all instances may obscure important differences in nutritional perspectives and management approaches between these groups. Eight users submitted multiple comments, which we included. Ultimately, we had no method to weigh the contributions of these repeated users.

Prospective investigations employing validated clinical instruments to evaluate nutritional status, eating behaviors, and interoceptive accuracy in broader FDS cohorts are sorely needed. Longitudinal work would help clarify whether structured nutritional counseling, particularly when it targets anxiety surrounding bodily sensations, yields measurable improvements in functional outcomes.

## 5 Conclusion

UwFDS articulate compelling, emotionally tinged connections between nutrition and their seizure experiences, with sentiment analysis revealing consistent positivity across all identified themes. We regard this positivity as a psychological artifact born of hope, reclaimed agency, and communal affirmation, not as evidence of direct metabolic influence on FDS pathophysiology. By embracing a psychologically mediated interpretation of nutritional concerns, clinicians can honor UwFDS’ lived realities while safeguarding diagnostic precision and averting iatrogenic reinforcement of inaccurate disease models. Integrating nutritional well‐being into comprehensive FDS care pathways is not only defensible but desirable, provided such integration remains steadfastly anchored in the functional nature of FDS.

## Author Contributions

**Hamid Abbasi**: writing – review and editing, writing – original draft, formal analysis, methodology, project administration, conceptualization, supervision, software, investigation, validation, visualization, resources, data curation. **Niloofar Taheri**: writing – original draft, investigation. **Ali Jafari**: writing – original draft, writing – review and editing, investigation, visualization, validation, methodology, resources, data curation, conceptualization.

## Funding

The authors have nothing to report.

## Ethics Statement

Informed consent was not procured due to the anonymity of Reddit users. However, this approach is generally deemed acceptable since Reddit (https://redditinc.com) operates as a public platform where users do not typically have an expectation of privacy.

## Conflicts of Interest

The authors declare no conflicts of interest.

## Supporting information




**Supplementary Material**: brb371497‐sup‐0001‐SuppMat.xlsx

## Data Availability

The data that support the findings of this study are available in the Supplementary Material of this article.
